# Overexpression approaches to advance understanding of *Candida albicans*

**DOI:** 10.1111/mmi.14818

**Published:** 2021-10-18

**Authors:** Laxmi Shanker Rai, Lasse van Wijlick, Murielle Chauvel, Christophe d’Enfert, Mélanie Legrand, Sophie Bachellier‐Bassi

**Affiliations:** ^1^ Unité Biologie et Pathogénicité Fongiques Institut Pasteur, Université de Paris, INRAE, USC2019 Paris France

## Abstract

*Candida albicans* is an opportunistic fungal pathogen that is responsible for infections linked to high mortality. Loss‐of‐function approaches, taking advantage of gene knockouts or inducible down‐regulation, have been successfully used in this species in order to understand gene function. However, overexpression of a gene provides an alternative, powerful tool to elucidate gene function and identify novel phenotypes. Notably, overexpression can identify pathway components that might remain undetected using loss‐of‐function approaches. Several repressible or inducible promoters have been developed which allow to shut off or turn on the expression of a gene in *C. albicans* upon growth in the presence of a repressor or inducer. In this review, we summarize recent overexpression approaches used to study different aspects of *C. albicans* biology, including morphogenesis, biofilm formation, drug tolerance, and commensalism.

## Graphical abstract

Overexpression approaches in *Candida albicans*. This review presents the development of overexpression strategies to understand different aspects of *C. albicans* biology, including morphological transitions, biofilm formation, drug resistance, and pathogenicity, using in vitro, ex vivo, or in vivo models, in individual assays or taking advantage of pooled collections.

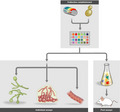

## 1 INTRODUCTION

Gene perturbation is the standard strategy to elucidate gene function and it can be achieved by both knockout and mis‐regulation approaches. The dramatic impact of aneuploidies in human and other model organism illustrates the importance of gene dosage for proper gene function (Sheltzer & Amon, 2011). In those instances, gene copy number variation without alteration to the basic genetic material is sufficient to cause a phenotype (Prelich, 2012; Tang & Amon, 2013). Naturally, molecular approaches aiming at either abolishing (knockout), reducing (knockdown), or increasing (overexpression) gene expression are commonly used by geneticists to decipher the role of a specific gene. The gene knockout strategy, used to understand the function of a gene through loss‐of‐function, faces several limitations: (a) many genes are expressed in a niche‐dependent manner, and thus the phenotypic effect linked to the absence of such genes can only be achieved in the specific conditions under which the gene is expressed; (b) the presence of functionally redundant genes will hamper the efficiency of such an approach when working with gene families; (c) the technical challenge of implementing knockout approaches in diploid organisms; and (d) only non‐essential genes can be studied by knockout approaches (unless using conditional knockout approaches). Alternatively, gene overexpression has been exploited by geneticists to understand biological pathways as a parallel approach to the loss‐of‐function approach. By altering cellular localization, complex stoichiometry or proper expression regulation, overexpression of a gene could result in a phenotype that would provide information about the gene function. This approach mimics gain of function mutations, complements loss‐of‐function phenotypes, and allows to study both essential and non‐essential genes (Prelich, 2012).

Genome‐wide ORF libraries represent useful resources for the implementation of overexpression approaches to elucidate gene function. In the past, genome‐wide overexpression libraries have been constructed for many model organisms, including *Arabidopsis thaliana, Caenorhabditis elegans, Drosophila melanogaster, Saccharomyces cerevisiae, Schizosaccharomyces pombe, Plasmodium falciparum, Xenopus laevis*, and *Escherichia coli* K‐12 using Gateway™ recombinational vectors (Bischof et al., 2013; Dricot et al., 2004; Gelperin et al., 2005; Gong et al., 2004; Grant et al., 2015; Rajagopala et al., 2010; Reboul et al., 2003).

*Candida albicans* is a diploid fungal pathogen lacking a complete sexual cycle, which limits the use of classical genetics. Efficient methods for the generation of homozygous null mutants have been developed and widely used for genetic analysis. However, overexpression tools can be an attractive alternative strategy for understanding gene function and for performing high‐throughput studies in this species. Indeed, constitutive promoters, such as P*_ACT1_* or P*_TDH3_*, are widely used in *C. albicans* to promote stable high‐level expression of the downstream gene (Delgado et al., 2003; Eckert & Mühlschlegel, 2009). In addition, several conditional promoters, such as P*_PCK1_*, P*_MAL2_*, and P*_MET3_*, have also been designed to regulate the expression of a *C. albicans* gene in specific growth conditions (Backen et al., 2000; Brown et al., 1996; Care et al., 1999; Eckert & Mühlschlegel, 2009; Leuker et al., 1997). Bacterial‐derived tetracycline‐responsive promoters have been adapted for *C. albicans*; their activity depends on tetracycline analogs, and they have been engineered to be either repressed (P*_TET_*_‐OFF_) or induced (P*_TET_*_‐ON_) upon addition of the compound, independent of the medium. These promoters have been used by our laboratory and other groups to generate collections of *C. albicans* P*_TET_*‐driven conditional overexpression strains that enable tetracycline derivatives‐dependent overexpression (Chauvel et al., 2012; Legrand et al., 2018; Park & Morschhäuser, 2005; Sahni et al., 2010).

Recently, the *C. albicans* ORFeome project resulted in the cloning of 83% of *C. albicans* ORFs in a Gateway™ donor vector (Legrand et al., 2018), constituting an unprecedented resource to stimulate the study of *C. albicans* biology by genome‐wide overexpression approaches.

Here, we review the development of various *C. albicans* overexpression collections and screens performed to give insights in different aspects of *C. albicans* biology, such as morphological transitions, biofilm formation, drug resistance, and gut colonization.

## 2 DEVELOPMENT OF DIFFERENT CONDITIONAL OVEREXPRESSION STRAINS OF *C. ALBICANS*

Overexpression approaches have been used in many model organisms, and several overexpression promoters have been developed to study gene function in *C. albicans* (Delgado et al., 2003; Eckert & Mühlschlegel, 2009; Leuker et al., 1997). In this section, we will describe the advancement of various overexpression tools to examine the different aspects of *C. albicans* biology.

Park and Morschhäuser (2005) adapted the bacterial tetracycline‐responsive (Tet) promoter for *C. albicans* by genetically fusing a modified version of the reverse Tet repressor from *E. coli* (modified to bind to the promoter and activate transcription upon binding with tetracycline or analogs) and the transcription activation domain of the Gal4 protein from *S. cerevisiae* (Park & Morschhäuser, 2005). To monitor the induction efficiency, the authors also placed a *C. albicans*‐adapted Green‐fluorescent protein‐coding reporter gene under the control of a Tet‐dependent promoter (P*_TET_*_‐ON_). By measuring the fluorescence levels, they demonstrated that gene expression could be efficiently achieved in *C. albicans* by addition of the tetracycline analog doxycycline independently of the cell morphology, for example, yeast, hyphae, and opaque forms. Further, they demonstrated the functionality of this promoter by being able to promote changes in cell morphology upon overexpression of *CDC42* and *NRG1*, regulators of *C. albicans* growth, and morphogenesis, respectively (Park & Morschhäuser, 2005).

Sahni et al. (2010) utilized this tetracycline‐dependent inducible promoter P*_TET_*_‐ON_ and constructed a total of 107 *C. albicans* overexpression strains (Table 1) encoding putative transcription factors regulating cell wall/membrane biogenesis, metabolism, adhesion, filamentation, and biofilm formation. The authors used the plasmid pNIM1 (Park & Morschhäuser, 2005) to produce the tetracycline‐dependent transactivator (Sahni et al., 2010).

**TABLE 1:** Overview of available overexpression libraries used in *Candida albicans*

Promoter	Vector (library size)	Composition of the library	Strain background	Condition screened	References
P*_TET_*_‐ON_	pNIM1 (107)	Transcription factors	P37005, MTLa/a	Sexual biofilm formation	Sahni et al. (2010)
P*_TET_*_‐ON_	pNIM6 (160)	Protein kinases	WO‐1, MTLα/α	White–opaque switching	Ramirez‐Zavala et al. (2013)
P*_TET_*_‐ON_	pNIM6 (222)	Protein kinases, protein phosphatases	WO‐1, MTLα/α	Hyphal morphogenesis	Bar‐Yosef et al. (2018)
P*_TET_*_‐ON_	pNIM1 pNIM6 (48)	Transcription factors	CAY616, MTLa/a	White–opaque switching	Lohse et al. (2016)
P*_ADH1_*	pZCF36DBH2 (82)	Transcription factors	SC5314	Fluconazole resistance	Schillig and Morschhäuser (2013)
P*_TET_*_‐ON_	CIp10‐P*_TET_*‐GTW (302)	Protein kinases, phosphatases, transcription factors and other	BWP17	Hyphal morphogenesis	Chauvel et al. (2012)
P*_PCK1_*	CIp10‐P*_PCK1_*‐GTW (277)	Protein kinases, phosphatases, transcription factors and other	BWP17	Hyphal morphogenesis	Chauvel et al. (2012)
P*_TET_*_‐ON_	CIp10‐P*_TET_*‐GTW (531)	Protein kinases, phosphatases, transcription factors and other	BWP17	Biofilm formation	Cabral et al. (2014)
P*_TET_*_‐ON_	CIp10‐P*_TET_*‐GTW (572)	Protein kinases, phosphatases, transcription factors and other	BWP17	Gut colonization fluconazole tolerance	Znaidi et al. (2018) Delarze et al. (2020)
P*_TET_*_‐ON_	CIp10‐P*_TET_*‐GTW (2,451)^a^	Genome‐wide	SN76		Legrand et al. (2018)

^a^ Still being developed.

Chauvel et al. (2012) constructed two *C. albicans* conditional overexpression strain collections by using the Gateway methodology (Walhout et al., 2000) which enables recombination‐based cloning of PCR‐amplified ORFs into a donor vector and their subsequent recombination‐mediated transfer into a variety of customized destination vectors (Chauvel et al., 2012). The authors constructed two conditional overexpression destination vectors, namely, CIp10‐P*_PCK1_*‐GTW‐TAP tag and CIp10‐P*_TET_*‐GTW, both derived from the *C. albicans* CIp10 integrative vector that can be systematically targeted at the *RPS1* locus (Chauvel et al., 2012) (Table 1). The CIp10‐P*_PCK1_*‐GTW‐TAP tag overexpression construct carries a Gateway cassette flanked by the gluconeogenesis‐induced *C. albicans PCK1* promoter and allows the fusion to a tandem affinity purification tag. The *PCK1* promoter is repressed when cells are grown in the presence of glucose and can be de‐repressed in the absence of glucose. CIp10‐P*_TET_*‐GTW contains the P*_TET_* promoter (Park & Morschhäuser, 2005) which is active in the presence of tetracycline derivatives. The CIp10‐P*_TET_*‐GTW constructs are developed with a unique barcode system enabling mixed pool experiments.

Cabral et al. (2014) further extended the *C. albicans* CIp10‐P*_TET_*‐GTW overexpression collection and built a 531‐strain collection allowing for overexpression of genes encoding transcription factors (180), protein kinases (72), protein phosphatases (34), protein related to DNA replication, recombination and repair (87), predicted cell surface proteins (61), and others (Cabral et al., 2014).

**FIGURE 1:**
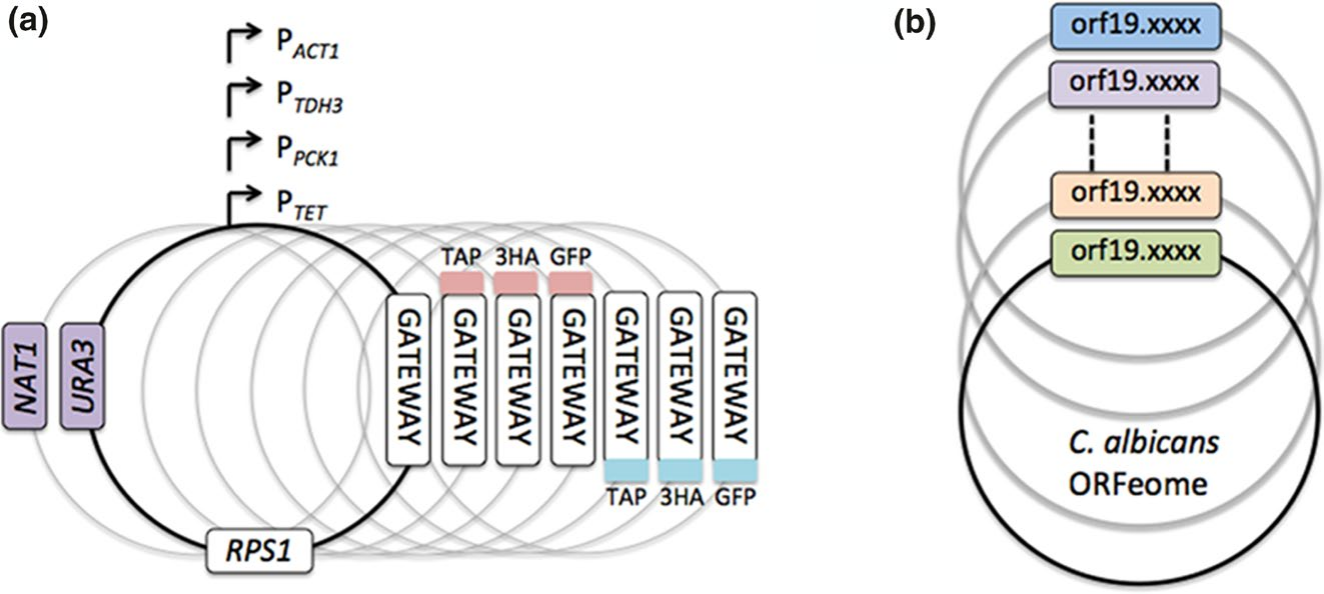
Tools that facilitate high‐throughput overexpression screens to understand the biology of *Candida albicans*. (a) A collection of 49 Gateway™‐adapted destination vectors for constitutive or conditional overexpression of untagged or tagged ORFs in *C. albicans*. (b) 83% of the *C. albicans* ORFeome has been cloned in the Gateway™ vector pDONR207 allowing subsequent transfer of the cloned ORFs to Gateway™‐adapted destination vectors

In the frame of the *C. albicans* ORFeome project 5,099 ORFs were cloned into a Gateway™ donor vector, representing 83% of the annotated coding sequences of *C. albicans* (Legrand et al., 2018). The authors also generated 49 expression vectors with different selection markers and promoters, allowing fusion with different tags. The ORFeome allows for the establishment of a genome‐wide collection of gene overexpression strains, a new resource for the functional genomic analysis in *C. albicans*. All information related to the ORFeome project are available on http://candidaorfeome.eu (Legrand et al., 2018) (Figure 1).

## 3 OVEREXPRESSION STUDIES TO UNDERSTAND *C. ALBICANS* BIOLOGY: THE CASE OF *C. ALBICANS* MORPHOGENESIS

*Candida albicans* is able to display distinct morphologies, such as yeast, hyphae, opaque, chlamydospore, and GUT (gastrointestinally induced transition) forms depending on different environmental cues (Noble et al., 2017; Pande et al., 2013; Sudbery et al., 2004). The yeast to hyphae transition is the most intensively studied as it is considered one of the major deciding virulence attributes during candidiasis (Jacobsen et al., 2012; Mayer et al., 2013). Along the years, standard gene knockout approaches have permitted the identification of signaling proteins, transcription factors, and effector molecules involved in the transition from yeast to hyphae (Kornitzer, 2019; Sudbery, 2011). However, recently developed overexpression strategies have given access to the role of additional proteins important for *C. albicans* morphogenesis which could not be identified with traditional knockout approaches.

**FIGURE 2:**
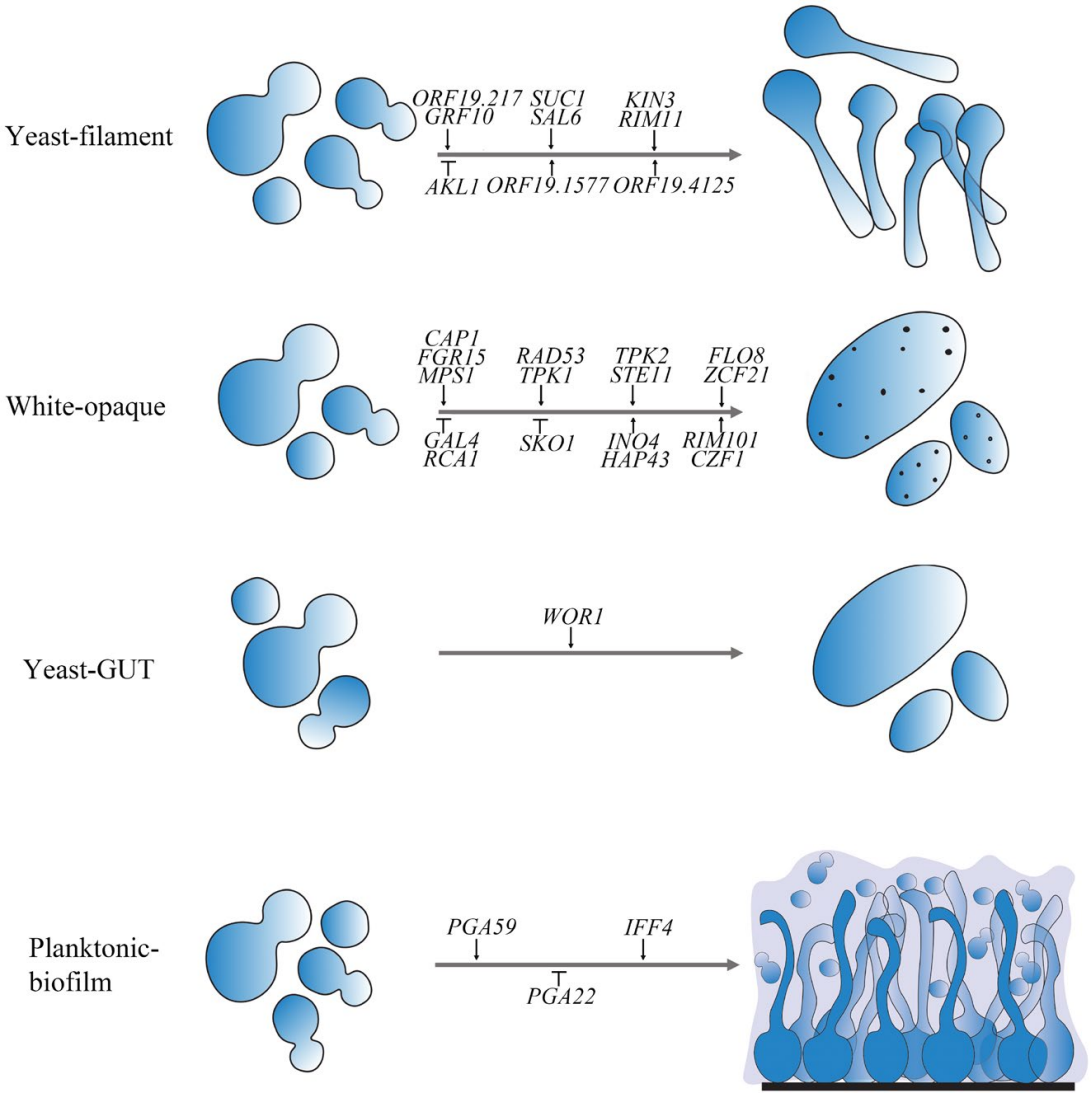
Schematic of gene overexpression studies affecting *Candida albicans* phenotypic transitions. Novel genes identified with overexpression studies during yeast‐filament, white–opaque, and yeast–GUT phenotypic transitions, or planktonic to biofilm formation are shown here

Several studies have made use of overexpression collections enriched with genes encoding protein kinases, protein phosphatases, transcription factors, and signaling proteins. Chauvel et al. constructed two overlapping *C. albicans* overexpression strain collections: in the first set, a total of 277 *C. albicans* genes were placed under the control of the P*_PCK1_* promoter, whereas in the second set, 302 genes were controlled by the stronger P*_TET_* promoter (Chauvel et al., 2012). In both collections, these overexpression cassettes were integrated at the *RPS1* locus of a *C. albicans* wild‐type strain. A total of 257 *C. albicans* genes are common between these two overexpression collections. Both collections were utilized to identify novel genes participating in *C. albicans* morphogenesis (Chauvel et al., 2012). The strains were first tested for the impact of gene overexpression on filamentous growth in liquid conditions which normally favor yeast growth. Eleven out of the 277 P*_PCK1_*‐dependent strains grown individually in YNB liquid medium with 2% casamino acids displayed a phenotype associated with pseudohyphae or hyphae formation at 30℃. These 11 genes encoded transcription factors (9), a protein kinase, and a protein phosphatase. Seven genes out of the 11 identified genes, namely, *CCN1, CAS5, FKH2, RFG1, SFL1, SFL2*, and *BRG1*, were already known to be involved in *C. albicans* morphogenesis (Chauvel et al., 2012). Interestingly, the two remaining candidates, *GRF10* and *ORF19.217*, encoding transcription factors, did not display any morphogenesis‐related phenotype when knockout strains were examined in different filamentation‐inducing media conditions (Homann et al., 2009). Similarly, *SAL6* and *SUC1* encoding a phosphatase and transcription factor, respectively, had never been linked to *C. albicans* morphogenesis. Thus, this screen with P*_PCK1_*‐driven overexpression strains uncovered a new set of genes involved in *C. albicans* morphogenesis. Then, Chauvel et al. also tested the 302 *C. albicans* P*_TET_*‐dependent overexpression strains in a liquid medium allowing P*_TET_* induction, without filamentation‐inducing cues. This screen revealed the involvement of 21 genes during *C. albicans* filamentation or pseudo‐filamentation, including *BRG1, SFL2, SFL1, RFG1, CAS5, FKH2*, and *ORF19.217* which were also uncovered in the first screen. However, some genes present in both collections, namely, *GRF10, SAL6*, and *CCN1*, did not display any phenotype when placed under the control of P*_TET_*. Interestingly, 14 additional genes triggered pseudo‐filamentation or filamentation, including *TEC1, EFH1, CPH1, PCL1, RAD53, SKN7*, and *STE11*, whose role in morphogenesis was previously uncovered either by knockout or overexpression approaches. This P*_TET_*‐driven overexpression screen also identified *RIM11* and *KIN3*, encoding protein kinases, which had not been previously associated with *C. albicans* morphogenesis. Interestingly, this P*_TET_*‐driven screen identified two uncharacterized genes, *ORF19.1577* and *ORF19.4125*, whose overexpression could cause filamentation, but the deletion mutants did not reveal a similar role during *C. albicans* filamentation (Chauvel et al., 2012) (Figure 2). Therefore, both screens suggested that overexpression studies can detect the involvement in morphogenesis of genes which are difficult to tackle by knockout approaches, possibly due to their niche‐specific expression.

Further, the authors also carried out a screen for filamentation with P*_TET_* overexpression strains on solid media and they could identify 17 genes whose overexpression altered the extent of *C. albicans* filamentation. Interestingly, the authors identified some genes, including *SFU1, GRF10*, and *ORF19.7227* (encoding a putative protein phosphatase inhibitor) which alter the filamentation behavior during overexpression specifically on solid media. Conversely, some genes, including *PLC1, RFG1, CAS5, RIM11*, and *ORF19.4125* showed alteration in filamentation only in liquid overexpression conditions.

This comparative study between the P*_TET_* and P*_PCK1_* overexpression strains for *C. albicans* morphogenesis revealed that the phenotypes obtained are also linked to the strength of the promoter. Taken together, these screens uncovered several previously unidentified genes, and thus indicated the potential role of overexpression approaches for *C. albicans* functional genomics.

P*_TET_*‐driven overexpression was also used to study the role of protein kinases and phosphatases during *C. albicans* morphogenesis (Bar‐Yosef et al., 2018). The authors extended a previously constructed overexpression library, based on vector pNIM1 (Park & Morschhäuser, 2005), composed of protein kinases and transcription regulators placed under P*_TET_*_‐ON_ (Ramirez‐Zavala et al., 2013) to a total of 222 genes (Bar‐Yosef et al., 2018). The authors performed a filamentation screen and identified the protein kinase Akl1, whose overexpression inhibits *C. albicans* hyphal elongation. They showed in parallel that the knockout mutant exhibited the opposite phenotype, that is, an acceleration of hyphal elongation under serum‐induced hyphal development.

Another type of cellular differentiation of *C. albicans* is the formation of large spherical cells, referred to as chlamydospores, when grown in adverse condition. However, this morphological state of *C. albicans* has not been studied up to the mark. An overexpression study of *RME1*, a zinc finger transcription factor identified its role during *C. albicans* chlamydospore formation. Indeed, overexpression of *RME1* led to the up‐regulation of chlamydospore‐related genes, and to the binding of a TAP‐tagged Rme1 at the promoters of these target genes (Hernandez‐Cervantes et al., 2020). Subsequent examination of the phenotype of the overexpressing strain in chlamydospore‐inducing conditions confirmed Rme1 involvement in the process, allowing a bypass of environmental cues and regulators that are required for chlamydospore formation (Hernandez‐Cervantes et al., 2020). As in the previous study, the knockout mutant showed the opposite phenotype, that is, the absence of chlamydospore formation, even in favorable conditions. However, very limited information is known about the regulatory circuits and effector molecules involved during the *C. albicans* chlamydospore development. Thus, we believe that utilizing the ORFeome collection of overexpression strains will help in identifying several key regulators and effector molecules of this under‐studied phenotypic state of *C. albicans*.

*Candida albicans* cells are also able to switch from a white yeast cell to a mating competent opaque cell. In the presence of pheromones, opaque cells utilize the transcription factor Cph1 and promote mating, whereas white cells undergo sexual biofilm formation via Tec1. Ramirez‐Zavala et al. (2013) utilized a collection of overexpression strains mainly composed of protein kinases to understand the signaling pathways operating during *C. albicans* white‐to‐opaque switching. In this overexpression screen, the authors identified the role of protein kinases Mps1, Rad53, Tpk1, Tpk2, and Ste11 during white‐to‐opaque switching (Ramirez‐Zavala et al., 2013). This study proposed that *STE11* overexpression induces white‐to‐opaque conversion without the α pheromones, and establishes an environment for rewiring where white cells recruit Cph1 instead of Tec1 and promote their transition to the opaque state (Ramirez‐Zavala et al., 2013).

Besides protein kinases, Lohse et al. (2016) systematically investigated the function of transcriptional regulators in the *C. albicans* white–opaque switch. In addition to a classical gene‐deletion approach, the authors used a set of 48 overexpression plasmids, based on pNIM1 and pNIM6 vectors (Park & Morschhäuser, 2005; Ramirez‐Zavala et al., 2013), allowing ectopic expression of selected transcription factors (Lohse et al., 2016). In contrast to other studies, only *MTL* ***a*** homozygous strains were used for the generation of the overexpression strain collection (Lohse et al., 2016). The core genetic network regulating the white–opaque switch is comprised of eight transcription factors, Ahr1, Czf1, Efg1, Ssn6, Wor1, Wor2, and Wor4 (Noble et al., 2017). Most central to the network are the two transcription factors Efg1, an inhibitor of the white–opaque switch and its antagonist Wor1, a positive regulator, promoting the white–opaque switch (Zordan et al., 2007). Ectopic expression of some of these previously identified regulators had already been used to gain insight into their functions; for instance, overexpression of *EFG1* and *AHR1* inhibits white‐to‐opaque switching (Sonneborn et al., 1999; Wang et al., 2011), while overexpression of *CZF1, WOR1, WOR3*, and *WOR4* stimulates white‐to‐opaque switching (Huang et al., 2006; Lohse et al., 2013; Lohse & Johnson, 2016; Vinces & Kumamoto, 2007; Zordan et al., 2006, 2007). Through the combination of gene deletion and gene overexpression, numerous other regulators could be identified by Lohse et al. (2016). Out of the 48 selected transcription factors, 12 affected the switching rates when overexpressed: eight stimulated white‐to‐opaque switching, including *CAP1, CZF1, FGR15, FLO8, HAP43, INO4, RIM101*, and *ZCF21*, while three genes (*GAL4, RCA1*, and *SKO1*) promoted opaque‐to‐white switching, and one gene, *ASH1*, affected switching in both directions (Lohse et al., 2016). For six of the 12 identified genes, the respective mutant also had a strong effect on the switching rate; however, gene deletion and gene overexpression did not always produce the opposite effect, reinforcing the notion that it is worth screening both types of libraries.

We believe that studying different morphological transitions, such as white to opaque, GUT cell induction (see below), or chlamydospore formation with newly generated P*_TET_*‐driven overexpression collections will bring a thorough understanding of these processes by which *C. albicans* adapt these phenotypes under specific environmental cues.

## 4 OVEREXPRESSION STUDIES TO UNDERSTAND *C. ALBICANS* BIOLOGY: THE CASE OF BIOFILM FORMATION

*Candida albicans* has the ability to form biofilms on tissues or implanted medical devices. Biofilms are composed of different cell types (yeast and filaments) which are encased in an extracellular matrix. The biofilm‐forming ability of *C. albicans* is considered as a major pathogenic attribute (Fanning & Mitchell, 2012). The biofilms formed by *C. albicans* are tolerant to conventional antifungal drugs, and thus make biofilm‐associated infections a clinical challenge (Fanning & Mitchell, 2012; Finkel & Mitchell, 2011). Key transcription regulators of biofilm formation have been identified and their function during *C. albicans* biofilm development has been studied by classical knockout approaches (Bonhomme et al., 2011; Fox et al., 2015; Nobile et al., 2012; Nobile & Johnson, 2015; Nobile & Mitchell, 2005). Similar studies revealed the role of chromatin modifiers on *C. albicans* biofilm formation; deletion of histone deacetylase Set3 core subunits (Hos2, Sif2, Snt1, and Set3), results in a rubbery biofilm, whereas a histone H3 variant (H3V^CTG^) negatively regulates *C. albicans* biofilm formation (Nobile et al., 2014; Rai et al.,  2019b). Alternative to the knockout approaches, Cabral et al. used a pooled collection of 531 doxycycline‐dependent barcoded strains allowing the overexpression of transcription factors, protein kinases and phosphatases, cell surface proteins and proteins related to DNA metabolism to identify novel genes involved during *C. albicans* biofilm formation in a microfermentor model (Cabral et al., 2014). The relative abundance of strains in the biofilms grown either in the presence or absence of doxycycline was assessed by barcoded microarrays, showing that the overexpression of 19 gene candidates resulted in an altered abundance without any significant alteration of their growth rates. Among these, 16 strains had an enhanced abundance, whereas three displayed reduced abundance in the multi‐strain biofilm. Surprisingly, the majority of these candidates encoded cell wall glycosylphosphatidylinositol (GPI)‐modified proteins, including Ihd1/Phg36, Phr2, Pga15, Pga19, Ppga22, Pga32, Pga37, Pga42, and Pga59 (Cabral et al., 2014). Interestingly, the phenotype upon overexpression did not affect filamentous growth, but only biofilm formation, and was for most of the strains only visible in a multi‐strain biofilm. Indeed, all the overexpression strains grew similar biofilms as the wild type when tested individually, but the *PGA59* and *PGA22* overexpression strains which developed more robust or thinner biofilms than the wild‐type reference strain, respectively (Figure 2). The authors further showed an increase in adherence properties upon overexpression of *IHD1/PGA36, PGA15, PGA22*, and *PGA59*. In contrast, overexpression of *PGA19, PGA32*, and *PGA37* resulted in reduced adherence to the surfaces (Cabral et al., 2014). Adhesive properties were also assessed in a conditional gene overexpression study (Fu et al., 2008). Here, the promoters of 25 genes encoding GPI‐anchored proteins were replaced by P*_TET_*_‐OFF_. The strains were tested for adherence to a plastic surface and *IFF4* was the only gene that modified adhesion properties upon overexpression, showing an increase. The effect of *IFF4* overexpression was then tested on epithelial cells and had a similar effect. In parallel, the study showed a decrease of adhesion to epithelial cells upon *IFF4* suppression (Fu et al., 2008).

Apart from pathogenic biofilms, *C. albicans* is also able to develop sexual biofilms in white cells homozygous for *MTL* ***a***. These cells are highly adhesive to plastic only in the presence of α pheromone. To understand the regulators of sexual biofilms, Sahni et al. examined a transcription factors overexpression collection for adhesion to plastic surfaces in the absence of α pheromone. Only one out of the 107 strains, overexpressing *TEC1*, could induce adhesion to the plastic surfaces in these conditions. Therefore, in this screen, authors identified *TEC1* as a key regulator for adhesion, regulating the downstream genes during *C. albicans* sexual biofilm formation (Sahni et al., 2010).

In our laboratory, about half of the ORFs available in the *C. albicans* ORFeome have now been transferred in an overexpression vector and introduced into *C. albicans* (Legrand et al., 2018). Ongoing screens for *C. albicans* biofilm formation with these ~2,500 overexpression strains indicated the involvement of protein kinases, phosphatases, and new regulators during *C. albicans* biofilm formation (Rai et al., 2019a). Further characterization of these sets of genes will bring insights in understanding the process of *C. albicans* biofilm development.

## 5 OVEREXPRESSION STUDIES TO UNDERSTAND *C. ALBICANS* BIOLOGY: THE CASE OF DRUG RESISTANCE AND TOLERANCE

Fluconazole is the most administered antifungal in clinical use against *C. albicans* and other *Candida* species, and increased resistance to azoles has become a serious problem. Known mechanisms leading to fluconazole resistance include altered drug uptake and efflux, and alterations of the ergosterol biosynthesis, which is the target of fluconazole (Robinson et al., 2017). In contrast, mechanisms underlying fluconazole tolerance are not well understood, and recent reports suggest that tolerance is related to slow growth of subpopulations of cells, allowing them to overcome drug‐induced stress (Rosenberg et al., 2018).

With the use of an overexpression collection of 572 *C. albicans* strains (Chauvel et al., 2012; Znaidi et al., 2018), Delarze et al. (2020) were able to identify two transcription factors encoding genes, *CRZ1* and *GZF3*, as well as *YCK2*, whose overexpression led to increased tolerance against fluconazole (Delarze et al., 2020). Crz1 functions downstream of the calcium‐mediated calcineurin signaling pathway and had already been implicated in fluconazole tolerance (Karababa et al., 2006; Onyewu et al., 2004). Additional evidence for involvement of calcium‐dependent calcineurin signaling was gained by transcriptional analysis of clinical isolates, indicating that fluconazole stimulated the calcineurin signaling pathway (Delarze et al., 2020). The GATA‐type transcription factor Gzf3 has not been studied in detail, but was shown to be activated by oxidative stress in a Cap1‐dependent manner (Wang et al., 2006). The *GZF3* ortholog in *S. cerevisiae* negatively regulates nitrogen catabolic gene expression (Soussi‐Boudekou et al., 1997), and interestingly, overexpression of *GZF3* resulted in slower vegetative growth rates (Sopko et al., 2006), which, in turn, could lead to increased drug tolerance (Rosenberg et al., 2018). The *YCK2* gene encodes the palmitoylated plasma membrane‐bound casein kinase I subunit, which is involved in glucose sensing and signaling in *S. cerevisiae* (Snowdon & Johnston, 2016), and contributes to hyphal morphogenesis in *C. albicans* (Alvarez & Konopka, 2007). Interestingly, inhibition of Yck2 results in increased susceptibility of *C. albicans* against caspofungin, belonging to the antimycotic class of echinocandins, suggesting that Yck2 might be an interesting target for the development of novel antifungal therapeutics (Blankenship et al., 2010; Caplan et al., 2020). The recurring identification of Crz1 demonstrates that the experimental approach is indeed robust, and that overexpression can be used as a complementary tool to knockout studies to explore mechanisms of drug tolerance and drug resistance.

In another approach, a complete library of artificially activated zinc cluster transcription factors was constructed and screened for increased fluconazole resistance (Schillig & Morschhäuser, 2013). A frequent cause of drug resistance is the acquisition of gain‐of‐function mutations in zinc cluster transcription factors leading to constitutive overexpression of their target genes (Coste et al., 2004, 2008; Flowers et al., 2012; Morschhäuser et al., 2007). For instance, in clinical isolates with an increased resistance to fluconazole, gain‐of‐function mutations were often found in *MRR1, TAC1*, and *UPC2*, encoding zinc cluster transcription factors (Schubert et al., 2011). The zinc cluster transcription factor family is unique to the fungal kingdom and consists of at least 82 regulators in *C. albicans* (Braun et al., 2005). In their novel approach, the authors fused each zinc cluster protein with the Gal4 activation domain under control of the constitutively active *ADH1* promoter, rendering it hyperactive (Schillig & Morschhäuser, 2013). Of note, overexpression of *TAC1* or *UPC2* alone from the *ADH1* promoter did not significantly affect fluconazole resistance; only overexpression of the Gal4 activation domain fusion proteins resulted in increased resistance, indicating that further signals are required to activate the transcription factors and that this requirement can be bypassed with the artificial fusion of the Gal4 activation domain (Schillig & Morschhäuser, 2013). Another explanation is that expression levels achieved with the *ADH1* promoter are not sufficient to cause hyperactivation of the protein. Apart from *MRR1, TAC1*, and *UPC2*, 14 other hyperactive transcription factors exhibited increased fluconazole resistance. While *MRR2, STB5*, and *ZNC1* conferred even higher fluconazole resistance than that caused by *UPC2* overexpression, the other regulators identified, comprising *CTA4, ARO80, AHR1, LYS14, LYS144, SUC1*, and the mostly uncharacterized transcription factors *ZCF2, ZCF9, ZCF25, ZCF35*, and *ZCF38*, conferred equally increased resistance levels. Detailed analysis of *MRR2* revealed that hyperactive Mrr2 causes activation of *CDR1*, encoding one of the major multidrug efflux pumps of *C. albicans* (Schillig & Morschhäuser, 2013).

This study makes an important contribution to the identification of underlying mechanisms that are subject to resistance, but also provides potential targets for therapeutic approaches. However, the study also shows that overexpression alone is sometimes not sufficient, but requires additional activation of the overexpressed protein, which can probably only be achieved in the context of a specific condition. Fusion of the Gal4 activation domain to the overexpressed protein is, therefore, a possibility to bypass this limitation and can be used to further gain insight into the regulatory networks of transcription factors.

## 6 OVEREXPRESSION STUDIES TO UNDERSTAND *C. ALBICANS* BIOLOGY: THE CASE OF *C. ALBICANS* GUT COLONIZATION

*Candida albicans* is a member of the human microbiota and commonly lives within the gastro‐intestinal (GI) and genital tracts of healthy individuals. Several studies have been performed to identify the regulatory network and effector molecules regulating *C. albicans* commensalism using knockout approaches in murine models (Chen et al., 2011; Noble, 2013; Perez et al., 2013; Rai et al., 2021; Witchley et al., 2021). An overexpression study has identified the role of the transcription factor *CRZ2* in *C. albicans* gut colonization. Znaidi et al. utilized a collection of 572 signature‐tagged P*_TET_*‐driven overexpression strains to identify new molecules important for *C. albicans* gut colonization. This overexpression collection, including genes encoding transcriptional regulators (183 ORF), kinases (77 ORF), phosphatases (33 ORF), cell wall (74) DNA replication/recombination, and repair genes (109), was first used to identify genes whose overexpression alters *C. albicans* fitness in vitro (Znaidi et al., 2018). The experiment was performed in a mixed pool to understand competitive fitness of these barcoded strains in the presence of doxycycline. The authors identified 25 genes belonging to DNA damage/cell cycle progression, hyphae formation, and signal transduction whose overexpression resulted in a decreased fitness (Znaidi et al., 2018). The authors then used this overexpression collection in a mixed pool to identify novel genes affecting gut colonization in a murine model. *CRZ2*, encoding a zinc finger transcription factor of the Cys2His2 family, showed an enhanced abundance under doxycycline‐mediated induction as compared to the uninduced condition. Conversely, knockout of *CRZ2* was associated with decreased abundance of *C. albicans* in the gastro‐intestinal tract. Genome‐wide binding of Crz2 revealed its role in modulating the expression of mannosyltransferase‐ and cell wall protein‐encoding genes. Thus, an overexpression screen identified Crz2 that modulates cell wall function during *C. albicans* gut colonization (Znaidi et al., 2018).

The most striking discovery with the overexpression approach is the identification of the GUT morphology of *C. albicans* which is triggered by overexpression of *WOR1*. Besides its role in the control of the white–opaque transition and mating (see above), *WOR1* proves to be a master regulator of *C. albicans* gut colonization as revealed by knockout approaches (Pande et al., 2013) (Figure 2). In the mammalian gut, the *WOR1* overexpression strain was shown to exhibit an altered colony morphology and transcriptome profile as compared to the yeast‐ or opaque‐cell types. These GUT cells rapidly dominate the mammalian gut over the yeast population after 10 days (Pande et al., 2013). Interestingly, this phenotype has not yet been observed in either *C. albicans* wild type or knockout mutants. Therefore, we believe that studying the ORFeome overexpression collection may reveal hidden morphologies of *C. albicans*, left undetected in wild type or knockout strains due to low frequency. Thus, the use of the ORFeome collection may identify novel regulatory networks and effector molecules of *C. albicans* commensal mode of growth.

In addition to these phenotypic transitions, many of the cell‐cycle‐regulating genes, in particular genes encoding for kinetochore proteins, are essential and their functions are often assessed using conditional mutants (Roy et al., 2011; Thakur & Sanyal, 2012). Therefore, an overexpression approach would allow to gain insight on the function of essential genes and disclose their mechanism of action. For these reasons, overexpression approaches are particularly well suited for investigating biological processes involved in genome stability. Our laboratory initially conducted an overexpression screen with a partial ORFeome to identify genes whose overexpression triggered genome instability in the form of increased loss of heterozygosity events (Loll‐Krippleber et al., 2015). In collaboration with the laboratory of Prof. Kaustuv Sanyal (Jawaharlal Nehru Centre for Advanced Scientific Research), we are now implementing this overexpression approach genome‐wide to identify new regulators of genome stability, such as kinetochore proteins, whose overexpression would alter genome stability or ploidy in *C. albicans*.

## 7 CONCLUSIONS AND PERSPECTIVES

Here, we have reviewed the application of overexpression approaches in identifying and understanding the phenotypic transitions and activities of the fungal pathogen *C. albicans*. Additional applications of overexpression approaches, for example, taking advantage of partial ORFeomes and Gateway‐adapted expression vectors have been reported for *Candida glabrata* (Schwarzmüller et al., 2014) and *Candida parapsilosis* (Pál et al., 2021). In most model organisms, including *C. albicans*, gene functions are often identified by knockout and/or knockdown approaches that are hindered in the case of diploid organisms, despite the recent adaptation of efficient gene editing using clustered regularly interspaced short palindromic repeats (CRISPR)‐Cas9 systems. In particular, the use of CRISPR‐Cas9‐dependent genome engineering in *C. albicans* and related species has proven useful in elucidating gene function and improving functional genomics (Min et al., 2016, 2018; Shapiro et al., 2018; Vyas et al., 2015). Yet, drawbacks in the use of stable knockout mutations is the widespread establishment of compensatory mutations (El‐Brolosy & Stainier, 2017), as well as the so‐called neighboring gene effect, where knocking out a gene interferes with the expression of a nearby gene, hence leading to erroneous gene functions. This phenomenon has been estimated to occur in 10% of *S. cerevisiae* knockouts (Ben‐Shitrit et al., 2012).

Although overexpression approaches are versatile, they also have their downside, such as toxic effects because of high levels of expression. Besides, endogenous levels of expression vary across genes with some genes showing low levels of expression and other genes showing high levels of expression. Hence, in systematic overexpression approaches as those we have described the level of overexpression achieved will vary from one gene to another and may, in the case of highly expressed genes, be insufficient to trigger a phenotype. Similarly, overexpression of single subunits of complexes might not have an effect.

However, overexpression approaches have many advantages. For instance, proteins can be ‘force‐activated’, bypassing the requirement for additional knowledge about activating signals. Thus, a gene can be associated with a phenotype using standard growth conditions, without any prior information about its function, while for knockout or knockdown mutants, the right conditions need to be found in order to test the phenotype. For instance, many transcription factors are expressed in a specific niche, hampering transcription network studies with knockout approaches. In contrast, forced expression of such transcription factors can be achieved through overexpression, allowing to gain insights in their role. Indeed, overexpression can be used to evaluate the impact on the transcriptome using transcript profiling, and to identify targets by Chromatin immunoprecipitation followed by sequencing (ChIP‐Seq) and ChIP‐MS (ChIP‐SICAP, Rafiee et al., 2016; van Wijlick et al., 2021). Overexpression approaches can, thus, help characterizing regulatory networks. Of note, overexpression approaches can generate off target binding of transcription factors, and thus a risk of getting false targets in the regulatory network. This limitation can be resolved by modulating the strength of promoters and/or the level of induction. In addition, it is possible to complement binding data with transcriptomic data to determine direct target genes.

As mentioned above, generating knockout mutants in *C. albicans* is a tedious process because of the diploid nature of this species but also the low efficiency of gene replacement approaches in *C. albicans*. Thus, generating genome‐wide collections of knockout mutants in different genetic backgrounds is nowadays beyond reach (a genome‐wide collection of knockout mutants in the laboratory strain SC5314 is still lacking). In contrast, the availability of a genome‐wide collection of overexpression plasmids allows to generate genome‐wide collections of overexpression strains relatively easily (if not tediously!) in different genetic backgrounds. Moreover, the barcoding of the overexpression plasmids allows pools of these plasmids to be assembled and transformed in any recipient strain. This allows implementing suppressor screens, the nature of the suppressor genes being rapidly determined using barcode sequencing in the transformants that show a suppressed phenotype. This approach was used successfully in our group in order to identify suppressors of the filamentation defect associated with the lack of the Yak1 kinase (Nesseir et al., manuscript in preparation) and could be expanded to the study of other biological processes in *C. albicans*. Of course, in large‐scale pool experiments with overexpression strains, the level of expression cannot be ascertained for each gene; there is thus no warranty of being exhaustive, as distinguished from deletion collections.

All in all, taking in consideration the respective advantages and drawbacks from both deletion and overexpression studies, we believe that using both knockout/knockdown and overexpression approaches would greatly improve our understanding in gene function. Therefore, we think these two approaches should be considered as complementary and used in parallel as often as possible.

## CONFLICT OF INTEREST

None.

## AUTHOR CONTRIBUTION

LSR, LvW, MC, CdE, ML, and SBB all participated in the preparation of the manuscript.
